# Does Previous Anaphylaxis Determine Differences Between Patients Undergoing Oral Food Challenges to Cow’s Milk and Hen’s Egg?

**DOI:** 10.3390/nu18020302

**Published:** 2026-01-18

**Authors:** Liliana Klim, Maria Michalik, Paweł Wąsowicz, Ewa Cichocka-Jarosz, Urszula Jedynak-Wąsowicz

**Affiliations:** 1 SSG at Children’s Diseases Clinic, Children’s University Hospital, Jagiellonian University Medical College, 31-008 Krakow, Poland; liliana.klim@student.uj.edu.pl (L.K.); maria02.michalik@student.uj.edu.pl (M.M.); 2 Independent Researcher, 30-389 Krakow, Poland; pjwasowicz@gmail.com; 3 Department of Pediatrics, Children’s University Hospital, Jagiellonian University Medical College, 31-008 Krakow, Poland; ewa.cichocka-jarosz@uj.edu.pl; 4 Department of Pulmonology, Allergy and Dermatology, Children’s University Hospital, 30-663 Krakow, Poland

**Keywords:** oral food challenge, food allergy, risk factors, history of anaphylaxis, specific IgE

## Abstract

**Background:** Oral food challenges (OFCs) are still the reference standard for confirming food allergy, yet the influence of previous anaphylaxis on challenge outcomes remains uncertain. Patients with a history of anaphylaxis are often considered at higher risk, which may affect the clinical decision-making process. This study aimed to identify predictors of OFC failure stratified by a history of anaphylaxis, given that prior investigations have predominantly considered anaphylaxis as an overall risk factor, without delineating distinct risk factor profiles according to anaphylaxis history. **Methods:** We conducted a retrospective evaluation of standard-of-care pediatric OFCs to cow’s milk and hen’s egg white. Eligible children had suspected or confirmed IgE-mediated allergy to cow’s milk protein (CMP) or hen’s egg white protein (HEWP) and were stratified by the presence or absence of previous anaphylaxis to the challenged food. Clinical data were compared between groups. Open OFCs were conducted under inpatient supervision with full emergency support. Logistic regression models were used to assess the relationship between comorbidities, specific IgE (sIgE) concentrations and OFC outcomes. Receiver operating characteristic (ROC) analysis evaluated diagnostic accuracy of sIgE concentrations in predicting OFC outcomes. **Results:** The analysis included 192 pediatric patients undergoing OFCs: 106 to CMP and 86 to HEWP. Six challenges (3.1%) were inconclusive, giving 186 valid results. The overall OFC failure rate was 32.3%. Patients with a past history of anaphylaxis more frequently underwent cow’s milk challenges (*p* = 0.01). Atopic dermatitis was a more common comorbidity in those without prior anaphylaxis (*p* = 0.04), regardless of the trigger. In hen’s egg challenges, children with a history of anaphylaxis reacted to significantly lower cumulative doses (*p* = 0.03) than patients without. Atopic dermatitis was identified as a predictor of OFC failure in children without prior anaphylaxis (*p* = 0.02), and asthma as a borderline predictor in those with previous systemic reactions (*p* = 0.05). Specific IgE concentrations correlated with OFC outcomes across allergens, with casein-sIgE showing the highest discriminative performance (AUC = 0.81) in children without previous anaphylaxis. **Conclusions:** Atopic dermatitis and asthma were identified as potential risk factors influencing OFC outcomes, depending on the patient’s history of anaphylaxis. The predictive accuracy of sIgE was different in groups stratified by presence of prior anaphylaxis, and the relationship between sIgE concentration and clinical reactivity was not identical across the two subpopulations. Casein-sIgE showed the highest diagnostic accuracy in children without previous severe reactions to CMP. Presence of anaphylactic reactions in the past is an important consideration when selecting children for OFCs to CMP and HEWP, since it delineates distinct risk factors for challenge failure in these patient populations.

## 1. Introduction

Food allergy (FA) represents a major and growing global health concern, with prevalence rates steadily increasing over the past decades [1,2,3]. This trend is particularly evident in children, among whom FA poses substantial challenges to health and quality of life [1,4,5,6]. In parallel, the incidence of food-induced anaphylaxis has risen markedly [7,8], as reflected by increasing hospitalization and emergency department admission rates worldwide [9,10]. According to the European Academy of Allergy and Clinical Immunology (EAACI), anaphylaxis is defined as “a severe, life-threatening generalized or systemic hypersensitivity reaction” [11]. Among pediatric patients, foods remain the predominant cause of systemic allergic reactions [12,13,14], including fatal cases [15]. Beyond their physical manifestations, FAs exert a profound psychosocial burden, significantly impairing quality of life and mental well-being, especially in individuals with a history of anaphylaxis [16].

The spectrum of allergens responsible for anaphylaxis varies by region and age, with cow’s milk, hen’s egg, peanuts, tree nuts, wheat, and shellfish representing the most common triggers [17,18,19,20]. Cow’s milk protein (CMP) and hen’s egg white protein (HEWP) are among the leading causes of food-induced anaphylaxis in early childhood [21,22,23]. Because strict allergen avoidance is difficult to maintain and often affects nutrition and psychosocial functioning, accurate diagnosis of FA is crucial to guide management and prevent recurrent reactions [24].

A range of diagnostic tools is available to identify food sensitization, including skin prick testing, serum allergen-specific IgE (sIgE) measurement, and component-resolved diagnostics (CRD) [25,26,27,28,29,30]. In addition, newer laboratory techniques—such as the basophil activation test (BAT) [31,32,33], mast cell activation test (MAT) [34,35,36], and assessment of eosinophil-related biomarkers (e.g., absolute eosinophil count or eosinophil-derived neurotoxin, EDN) [37,38]—provide valuable supplementary information. Nevertheless, the oral food challenge (OFC) remains the gold standard for confirming FA due to its unmatched diagnostic accuracy and clinical relevance [26,39,40,41]. Beyond diagnosis, OFCs allow evaluation of tolerance thresholds, assessment of the effect of food processing on allergenicity [42], and initiation of oral immunotherapy (OIT) [43,44], which is currently recognized as an effective active treatment strategy [45,46].

OFCs with CMP and HEWP are routinely performed as the cornerstone of FA diagnosis and management in pediatric populations [47,48,49,50,51,52,53,54]. However, the indications for OFC vary widely, resulting in substantial heterogeneity among tested patients [55,56,57,58]. These range from children with a documented history of anaphylaxis to those who have experienced only mild or no previous symptoms [59,60,61]. Moreover, children with atopic dermatitis or other allergic conditions are frequently tested for food sensitization despite lack of previous exposure or reaction, predisposing to overdiagnosis of FA [60,62]. Prior anaphylaxis has been identified as a major risk factor for OFC failure [63,64,65,66,67], underscoring the need for individualized risk assessment and specific safety precautions during the procedure. Consequently, children with and without a history of anaphylaxis undergoing OFCs represent two distinct clinical populations [64].

Given these differences, separate consideration of these two groups may enhance both clinical assessment and research analyses. The aim of this study was to compare children with and without a prior history of anaphylaxis to the challenged food, undergoing OFCs to CMP and HEWP, and to explore pre-existing differences between these groups. We further sought to identify predictors of OFC failure stratified by anaphylaxis history. To our knowledge, this is among the first studies to analyze OFC outcomes according to prior anaphylaxis, providing a framework for optimizing OFC protocols and minimizing unnecessary challenge failures in the future.

## 2. Materials and Methods

### 2.1. Study Design

The present retrospective analysis included standard-of-care oral food challenges (OFCs) performed between 1 January 2014 and 31 July 2025 in the Pediatric Allergy Department. The study was based on chart review of OFCs conducted with cow’s milk and hen’s egg white. Eligible patients were either suspected or diagnosed with IgE-dependent food allergies (FAs) to these two allergens. OFCs for food protein-induced enterocolitis (FPIES) or other non-Ige-mediated disorders were excluded. Clinical data, including patients’ gender, age, allergic comorbidities (asthma, atopic dermatitis, multi-food allergy), as well as a family history of atopy, were gathered from the databases of the department. Information on history of anaphylaxis was obtained from records of the Allergy Department, the Emergency Department and medical documentation provided by parents or guardians. The included patients were divided into two groups according to the presence of history of anaphylaxis to the challenged food. OFC outcomes and clinical characteristics were analyzed separately in the two patient groups, and intergroup differences were assessed.

Stringent observance of ethical standards and full compliance with the principles enshrined in the Declaration of Helsinki was ensured in the course of the study. Institutional ethics committee approval was obtained for this study (118.0043.1.274.2025). The requirement for informed consent was waived due to the retrospective nature of the study. Written informed consent for the OFC itself was obtained from parents or legal guardians of the patients prior to each procedure.

### 2.2. Specific IgE Testing

A test to determine specific IgE (sIgE) concentrations was conducted as part of the process of evaluating the patient’s eligibility for an OFC. Measurement of sIgE concentrations was performed using the ImmunoCAP^®^ Specific IgE assay (Phadia, Uppsala, Sweden). Values exceeding 0.35 kU/L were considered positive. All results were obtained not later than six months prior to the challenge date. For subsequent statistical analyses, sIgE titers above 100 kU/L were capped at 100 kU/L. Component-resolved diagnostics (CRD) were unavailable for a subset of patients, as part of the cohort underwent OFCs before CRD became routinely implemented in clinical practice in the Allergy Department. Furthermore, CRD testing was deemed unnecessary in some children displaying low allergen extract-based sIgE values. Moreover, in some patients sIgE were not available, as they were qualified for an OFC based on a skin prick test.

### 2.3. Oral Food Challenges

Open oral food challenges (OFCs) were based on previously described challenge protocols [68] and performed in accordance with AAAAI–EAACI PRACTALL guidelines [39,56]. Eligible children were in good health, with comorbidities controlled and interfering medications withheld [69]; tests were not scheduled during relevant pollen seasons. All procedures were conducted in an inpatient setting under supervision of a physician experienced in pediatric anaphylaxis, with access to medications and full resuscitation support available.

Briefly, food was administered in incremental doses every 30 min to a cumulative, age-appropriate serving (e.g., 200 mL milk, 1 egg); baked milk and baked egg challenges corresponded to 32 mL milk in one large or two small muffins and one-third of a large egg in one large or two small muffins, respectively. Non-baked CMP challenges involved fresh or fermented milk and modified infant formula, while non-baked HEWP challenges consisted of ingestion of a hard-boiled egg. Children were monitored during dosing and for at least 4 h following completion of the challenge or until signs of clinical reactivity subsided for those patients who failed the challenge. OFCs were discontinued if objective allergic symptoms or concerning persistent subjective complaints occurred [69,70]. Outcomes were classified as negative (passed) when the full dose was tolerated, or positive (failed) when symptoms of allergic reaction occurred, with severity graded according to Sampson’s scale [71]. In cases where the patient and/or parent refused to continue the challenge in the absence of symptoms, results were considered inconclusive, and a maintenance of the elimination diet was recommended. The choice of baked versus raw allergen was guided by clinical history, prior anaphylaxis and reaction severity, sIgE concentrations, as well as study objectives, with higher-risk patients initially challenged with baked forms.

### 2.4. Statistical Methodology

All statistical analyses were performed with STATISTICA 13.3 (StatSoft, Tulsa, OK, USA, licensed under Jagiellonian University), and Python 3.12 (Python Software Foundation, Wilmington, DE, USA) with the pandas, numpy, scipy, scikit-learn, and matplotlib libraries (https://pypi.org/, accessed on 12 September 2025).

Continuous variables were summarized as medians, means, ranges, and interquartile ranges (IQRs), while categorical variables were expressed as counts and percentages. Intergroup comparisons were conducted using the Pearson chi-square test for categorical data and the Mann–Whitney U test for continuous variables. A two-tailed *p* value < 0.05 was considered statistically significant.

Regression analysis: To examine the relationship between sIgE concentration and the probability of oral food challenge (OFC) failure, logistic regression models with a sigmoidal link function were fitted. Specific IgE concentrations were log-transformed to account for their skewed distribution. Model parameters were estimated using non-linear least squares optimization (curve_fit, SciPy).

The logistic model maps sIgE concentrations onto a probability scale (0–1) and captures the threshold-like increase in clinical reactivity with rising sIgE values. The inflection point of the fitted curve corresponds to the sIgE concentration associated with approximately 50% probability of OFC failure, providing a clinically interpretable threshold.

To empirically validate the model, sIgE concentrations were divided into five equal-sized bins (quintiles) within each subgroup (with and without a history of anaphylaxis). For each bin, the mean sIgE concentration and observed proportion of OFC failures were calculated and plotted as empirical points over the fitted curves, enabling visual comparison of observed and predicted probabilities.

ROC analysis: To assess the diagnostic performance of individual sIgE components in predicting OFC outcomes, Receiver Operating Characteristic (ROC) curves were generated.

The area under the curve (AUC) was calculated as a global measure of discriminative performance. For each component, the optimal threshold was determined using Youden’s index, which maximizes the sum of sensitivity and specificity. At this threshold, sensitivity, specificity, and the corresponding sIgE concentration were reported. Analyses were performed separately in subgroups with and without a history of anaphylaxis.

## 3. Results

### 3.1. General Information and Patient Demographics

A total of 192 patients who underwent oral food challenges (OFCs) to cow’s milk (106) or hen’s egg white (86) were included in the study. The group of patients suspected of having FAs comprised individuals with a history of frequent atopic dermatitis exacerbations, pre-existing sensitizations, or other symptoms mimicking those typically associated with FAs. In six cases (3.1%), the OFC results were deemed inconclusive due to patient’s refusal to consume the challenged food, including four patients with a history of anaphylaxis to the challenged food and two without. All inconclusive results were recorded in children under 3 years of age. These patients were accounted for in the overall population characteristics but were excluded from subsequent statistical analyses, as indicated in the corresponding tables. Finally, complete data were available for 186 challenged patients and included in the analysis.

The characteristics of the study population are summarized in Table 1. In patients with a history of anaphylaxis to the challenged food, prior reaction severity was evenly distributed across grades 2, 3, and 4. Only two patients (1.8%) had experienced grade 5 anaphylactic shock, with CMP identified as the trigger in both cases.

The overall failure rate across all OFCs was 32.3% (62 cases). Of these, 32 patients (30.2%) failed the cow’s milk protein (CMP) challenge and 30 (34.9%) failed the hen’s egg white protein (HEWP) challenge. The characteristics of the study population by the challenged allergen (CMP or HEWP) are presented in Table S1, attached to Supplementary Materials.

### 3.2. Comparison Between Patient Groups by History of Anaphylaxis to the Challenged Food

In line with the study objective, participants were stratified according to the presence or absence of a history of anaphylaxis to the challenged food. The characteristics of these two groups are summarized in Table 2. Challenges involving CMP were performed significantly more often in patients with a history of anaphylaxis compared with those without (63.6% vs. 43.9%, *p* = 0.01). In contrast, atopic dermatitis was more prevalent among patients without a history of anaphylaxis than among those with such a history (70.7% vs. 56.4%, *p* = 0.04). There were no other statistically significant differences between the groups. While a history of anaphylaxis did not generally predict a higher risk of severe reactions during failed OFCs, we observed one patient with prior anaphylaxis who developed anaphylactic shock during the procedure. Notably, none of the patients without a history of anaphylaxis experienced reactions of comparable severity.

As described in the Materials and Methods section, OFCs were performed using baked or non-baked forms of CMP and HEWP. Baked food predominated in both CMP and HEWP challenges, irrespective of patients’ history of anaphylaxis. Baked CMP was administered in 46 OFCs (65.7%) performed in children with a history of anaphylaxis and in 27 OFCs (75.0%) among those without such a history (*p* = 0.33). Similarly, baked HEWP was used in 27 OFCs (67.5%) conducted in patients with a history of anaphylaxis and in 34 OFCs (73.9%) in those without (*p* = 0.51).

The comparison of cumulative reactive doses of allergenic protein in failed CMP and HEWP OFCs, stratified by prior history of anaphylaxis to the challenged food, is presented in Table 3. A statistically significant difference was observed for HEWP challenges, with patients who had a history of anaphylaxis reacting to lower cumulative doses than those without such a history (*p* = 0.03). No significant difference in cumulative reactive dose was found for CMP challenges, nor between CMP and HEWP challenges within either anaphylaxis subgroup.

### 3.3. Factors Influencing OFC Outcome in Studied Groups

Potential predictors of OFC outcomes were examined using logistic regression to assess the influence of clinical and demographic variables. The estimated effects of these variables on OFC results are presented in Table 4. Among children without a history of anaphylaxis to the challenged food, atopic dermatitis was significantly associated with OFC failure (*p* = 0.02). In contrast, this association was not observed in participants with prior anaphylaxis. In children with a history of anaphylaxis, asthma appeared to be a potential risk factor for a positive OFC outcome (*p* = 0.05). A family history of atopy demonstrated a trend toward significance among children without previous anaphylaxis (*p* = 0.06), similarly to multi-food allergy in the same patient group (*p* = 0.07). No other clinical or demographic variables significantly influenced OFC outcomes.

### 3.4. Specific IgE Concentration as a Predictor of OFC Outcome in Studied Groups

Specific IgE (sIgE) values for CMP extract were available in 99 patients, with casein-specific sIgE values available in 85 cases. For HEWP, extract sIgE values were measured in 77 patients, and ovomucoid-specific sIgE values were accessible in 58 cases. In some children, sIgE values were not obtained due to reasons specified in the Materials and Methods section.

Specific IgE concentrations for passed and failed cow’s milk OFCs in patients with and without a history of anaphylaxis to the challenged food are presented in Table 5. A statistically significant difference was observed between CMP-specific IgE median concentrations in passed and failed OFCs in both groups, regardless of anaphylaxis history. A similar association for casein-specific IgE concentrations was noted only among patients without a history of anaphylaxis. No substantial differences in CMP- or casein-specific IgE values were observed when comparing patients with and without anaphylaxis history, for either passed or failed challenges.

Comparative analyses analogous to those described above were conducted for hen’s egg white OFCs, with the corresponding results summarized in Table 6. Median HEWP-specific IgE concentrations differed significantly between passed and failed OFCs in both groups, irrespective of anaphylaxis history. In contrast, a similar association for ovomucoid-specific IgE concentrations was observed exclusively among patients with a prior history of anaphylaxis. Notably, in passed challenges, both HEWP- and ovomucoid-specific IgE concentrations were significantly higher in patients without a history of anaphylaxis compared with those who had previously experienced anaphylaxis to the tested food. No significant intergroup differences were detected for failed challenges.

**Figure 1:**
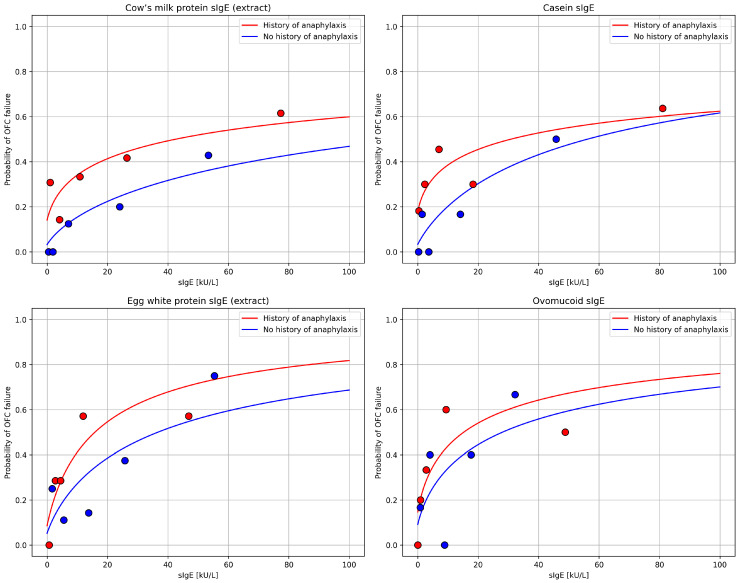
Sigmoid-fitted probability curves. sIgE—specific IgE; OFC—oral food challenge.

When outcome probabilities were analyzed, the proportion of positive OFCs increased with rising sIgE concentrations to CMP (extract), casein, HEWP (extract), and ovomucoid. The corresponding sigmoid-fitted probability curves are shown in Figure 1.

**Figure 2:**
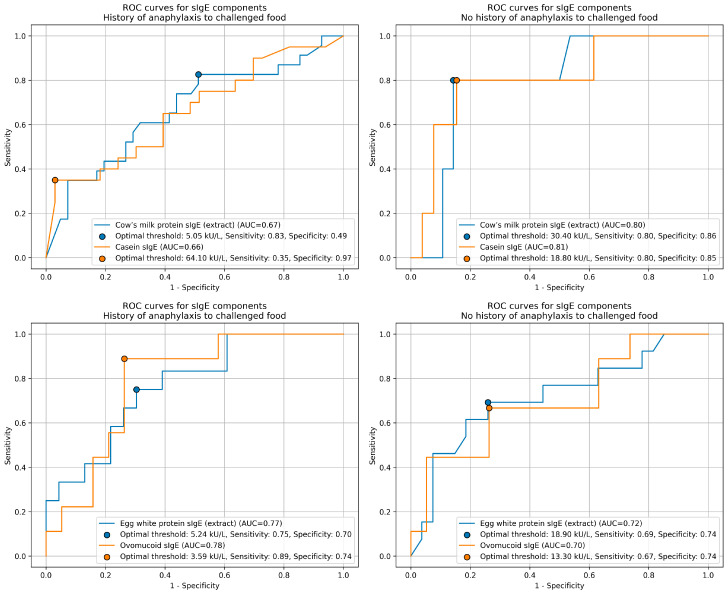
Receiver operating characteristic (ROC) curves for cow’s milk and hen’s egg sIgE concentrations. sIgE—specific IgE; AUC—area under the curve.

Receiver operating characteristic (ROC) curve analysis was performed to determine the most optimal cutoff sIgE concentrations to predict outcomes of OFCs to cow’s milk and hen’s egg white with consideration for history of anaphylaxis to the challenged food. Cutoff points were chosen to ensure both OFCs’ safety by maximizing specificity while maintaining an acceptable Youden’s index. ROC curves, with the optimal thresholds for all variables, their respective area under the curve (AUC), sensitivity and specificity values are presented in Figure 2.

For most ROC analyses, the AUC values exceeded 0.70, indicating acceptable to moderate discriminative ability (HEWP- and ovomucoid-sIgE in both groups stratified by anaphylaxis history, and CMP-sIgE in the non-anaphylaxis group). Among all tested parameters, casein-sIgE in the group without a history of anaphylaxis showed the highest discriminative performance (AUC = 0.81). In contrast, the AUCs for CMP- and casein-sIgE in patients with a history of anaphylaxis were lower (0.67 and 0.66, respectively), indicating limited predictive utility.

## 4. Discussion

An OFC remains the gold standard for diagnosing FA, since it provides most accurate and clinically relevant results [26,39,40,41], which are particularly important in children so as to prevent unnecessary elimination diets and nutritional deficiencies [72,73,74]. OFCs are performed in two principal groups: patients with and without a history of anaphylaxis. The latter often includes children tested for food sensitization due to AD, recurrent infections, family history of atopy, or parental concern [62,75,76]. Sensitization to CMP and HEWP is common [6], and given their nutritional importance in early childhood [77,78,79], confirming clinical allergy is essential to counteract unnecessary food avoidance [80,81,82,83].

### 4.1. General Information and Patient Demographics

In all analyzed groups, OFCs were predominantly performed using baked products. The majority of children with milk or egg allergy tolerate the baked form of these foods [84,85]. It is therefore common practice to initially perform an OFC with a baked product, followed by a subsequent challenge with the unbaked form. Tolerance to baked food often indicates an impending resolution of allergy to the unheated form [86,87,88,89], as explained by the reduced allergenicity of thermally processed foods, resulting from protein denaturation at elevated temperatures [84,90]. Furthermore, the incorporation of baked milk and egg into the diet is considered beneficial, functioning as a form of oral immunotherapy [91], with gradual increases in the amount and reduction in the degree of thermal processing promoting the acquisition of full tolerance [24,84,92,93,94,95,96].

As previously reported [54,64,97,98,99], our cohort showed a predominance of male patients, with 127 (over 66%) procedures performed in boys. Males tend to exhibit higher total and allergen-specific IgE (sIgE) concentrations than females, which correlates with an increased prevalence of FAs [100] and a higher incidence of food-induced anaphylaxis [14,20]. These differences may contribute to higher frequency of severe reactions and higher failure rates among male patients undergoing OFCs [55,101]. Moreover, before puberty, boys are generally more susceptible to atopic diseases than girls [102], which may be explained by the tendency of males to develop a more pronounced type 2 immune response, predisposing them to enhanced allergic sensitization and, consequently, a greater need for OFCs in this population. In contrast, females typically display a more balanced type 1/type 2 immune profile [103].

In our analysis of 192 OFCs, the overall failure rate was 32.3% (N = 62), which is consistent with previously reported rates ranging from 8.7% to 85% [54,67,97,104,105,106]. Notably, all inconclusive challenges (N = 6, 3.1%)—cases in which patients refused to ingest the tested food—occurred in children under three years of age. This finding highlights the challenges of performing OFCs in the youngest age group, as food refusal is common among infants and toddlers, even in good health [107]. In our study, the median time to symptom onset was 90.0 min in both groups, which underscores the importance of close, continuous monitoring from the outset of the challenge, irrespective of anaphylaxis history.

### 4.2. Comparison Between Patient Groups by History of Anaphylaxis to the Challenged Food and Factors Influencing OFC Outcome in Studied Groups

Since a history of anaphylaxis has been identified as a risk factor for OFC failure [63,64,65,66,67], one of the objectives of our study was to compare patient populations undergoing OFCs that differed with respect to anaphylaxis history. In our cohort, OFCs to CMP were more frequently performed in children with a previous history of anaphylaxis than in those without. CMP appears to exhibit greater allergenic potency than HEWP, as observed by Cichocka-Jarosz et al. [21], who reported that lower reactive doses of CMP than HEWP were capable of inducing anaphylaxis. Similarly, Valluzzi et al. [108] found that milk caused reactions at lower doses than hen’s egg in children. Moreover, CMP more commonly provokes lower respiratory symptoms, including respiratory failure [109,110]. Taken together, these findings suggest that children allergic to CMP are more likely to present with a broader allergic phenotype, including a higher incidence of anaphylaxis.

A higher prevalence of AD was observed among children without previous anaphylactic episodes compared with those with a history of anaphylaxis. This may be interconnected with the fact that individuals with chronic skin inflammation are often tested for food sensitization, including serum sIgE panels, even without clear evidence of prior reactions [62,75,76,111].

Moreover, AD was found to increase the risk of OFC failure in patients with no anaphylaxis history, paralleling higher AD prevalence in this group, as previously reported by other authors [64,104,112]. An association between AD and FA reflects that food allergens may trigger or exacerbate cutaneous symptoms in sensitized patients [113,114]. Although not every patient with AD requires extensive FA diagnostics, such evaluation may be justified when food allergens appear to worsen cutaneous symptoms [111,113]. The coexistence of AD and FA likely reflects shared pathogenic mechanisms, particularly skin barrier dysfunction and type 2 inflammation [115]. Importantly, while food sensitization is common in AD, confirmed IgE-mediated FA is observed less frequently, with risk correlating primarily with the severity and chronicity of AD [111,114]. It is worth noting that AD plays a role in the FA causality pathway and not vice versa. Furthermore, only OFCs can reliably confirm FA [113]. Recent evidence discourages routine sensitization testing and elimination diets in most patients with AD [114]. Additional diagnostics may be beneficial in infants or patients with severe, treatment-resistant disease. Nevertheless, clinicians should consider the risks of false-positive results and unnecessary dietary restrictions [113].

An association between a family history of atopy and an increased prevalence of FA in offspring has been previously reported [116,117,118]. Although the results did not reach statistical significance, a suggestive non-significant association between family history of atopy and OFC failure was observed among children without a prior history of anaphylaxis (*p* = 0.06). A similar pattern was noted for multiple FAs within this subpopulation (*p* = 0.07). Previous studies have identified multi-food allergy as a risk factor for OFC failure in certain cohorts [104]. Allergic multimorbidity is common in children undergoing OFCs [109,119,120]. Supplementary FA testing might be therefore beneficial in individuals with a family history of atopy or pre-existing FAs. However, further investigation in larger, well-characterized cohorts is needed.

None of the aforementioned variables were identified as risk factors for OFC failure among patients with a history of anaphylaxis. In contrast, although asthma did not reach statistical significance in this subgroup, a suggestive association was observed (*p* = 0.05). This potential relationship could become significant in a larger cohort, warranting further investigation. Asthma has been reported to increase the likelihood of OFC failure in certain populations [97,112], including findings from our previous study [68], where asthma was identified as an important risk factor for failing an OFC in a general pediatric cohort. Moreover, it may provoke more severe reactions during failed challenges [54,121,122], as lower respiratory symptoms are frequent among asthmatic patients [109,110,123]. Notably, asthma is a common comorbidity among children who experience food-induced anaphylactic reactions [14,15,22]. Patients with asthma are also more likely to develop severe allergic reactions, including fatal anaphylaxis [15,23,123,124]. Although some studies have failed to confirm these associations [125,126], a strong link between asthma and FAs has been established, with approximately 48% of patients with asthma also being affected by FAs [127]. IgE-mediated FAs, particularly to CMP and HEWP, often coexist with asthma or precede its onset as a part of the “atopic march” [128,129]. Given the divergent findings in the literature [127], further research is required to clarify the true relationship between asthma and the risk of OFC failure or symptom severity.

Although a history of anaphylaxis has previously been identified as a risk factor not only for challenge failure but also for increased symptom severity during failed OFCs [65], our findings did not replicate these associations. In our cohort, failure rates were comparable between the two anaphylaxis groups, and severe symptoms were not more prevalent among participants with a history of anaphylaxis, similarly to observations from a case series reported by Honda et al. [101]. These results suggest that a history of anaphylaxis alone might not independently increase the risk of OFC failure or more severe reactions. Instead, it may interact with other, varying risk factors that—when present—could influence these outcomes, depending on anaphylaxis history. However, it is essential to note that this finding is derived from a limited number of severe cases and should be interpreted with caution, highlighting the need for further investigation in larger cohorts.

### 4.3. Specific IgE Concentration as a Predictor of OFC Outcome in Studied Groups

Elevated sIgE concentrations are associated with an increased risk of challenge failure [130]. In recent years, component-resolved diagnostics (CRD) have emerged as a valuable tool in diagnosing FAs [28]. Several studies have demonstrated that sIgE concentrations to allergen extracts and components differ significantly between patients with passed and failed OFCs or severe reactions [61,63,98,130,131,132]. In our cohort, among patients for whom serological data were available, sIgE concentrations were generally higher in children who failed an OFC compared with those who passed, similarly to findings from previous studies [63,98,112,133]. The risk of OFC failure was rising with increasing sIgE concentrations across most examined subgroups, including those stratified by history of anaphylaxis and by allergen type (CMP or HEWP). The only exception, probably due to a low sample size, was ovomucoid-specific IgE in patients without a history of anaphylaxis to HEWP, where the difference was not statistically significant. In children with a history of anaphylaxis to CMP, casein-specific IgE concentrations approached statistical significance (*p* = 0.05).

We also aimed to compare sIgE concentrations between patients with and without a history of anaphylaxis. In passed challenges, both HEWP- and ovomucoid-specific IgE concentrations were significantly higher in patients without prior anaphylaxis compared with those who had previously experienced anaphylaxis to hen’s egg. Simultaneously, children with a history of anaphylaxis to HEWP reacted to nearly threefold lower median cumulative doses of allergenic protein than those without prior anaphylaxis. No such associations were observed for CMP, nor between the reactive doses of CMP and HEWP. However, the propensity of CMP to elicit allergic reactions at lower doses than HEWP is commonly known [21,108]. Our findings suggest a higher tolerability threshold in children without previous anaphylaxis to HEWP despite higher sIgE concentrations. Similarly, a review by Yanagida et al. [131] demonstrated that a history of food-induced anaphylaxis was related to low threshold doses and occurrence of severe reactions during the OFC. However, in a study by Sasaki et al. [134], patients with higher sIgE concentrations (≥100 UA/mL) had significantly lower total loading dose than the <100 UA/mL group. Considering these divergent results, a history of anaphylaxis may reflect a lower threshold of reactivity rather than higher sensitization levels, indicating a possible dissociation between sIgE concentration and clinical reactivity, which has been previously observed [29]. Clinicians should also be aware that low sIgE concentrations are not always a marker of tolerance, especially in children with prior anaphylaxis, who frequently avoid the offending food for a long period, which does not facilitate induction of tolerance. These children are at risk of OFC failure, or even anaphylaxis during the procedure despite relatively low sIgE concentrations, which may be lower than in patients with no such strict avoidance [135]. Given the observed differences for HEWP, it may be feasible to initiate OFCs in children without a history of anaphylaxis using higher starting doses than in those with prior anaphylaxis, without compromising the safety of the procedure. Further studies are warranted to clarify these relationships for CMP.

Although sIgE is not a fully reliable marker for FA, as previously noted by several authors [136,137,138], practical tools are needed to guide OFC eligibility. Multiple studies have proposed a wide range of serum sIgE cutoff values to predict outcomes of OFCs to cow’s milk and hen’s egg. For CMP, suggested thresholds vary considerably, from as low as 0.30 kU/L to as high as 33.90 kU/L [24,40,67,104,130,139,140,141,142]. For HEWP, reported cutoff levels range from 0.94 kU/L to 10.70 kU/L [24,40,67,104,130]. Within CRD, casein (Bos d 8) and ovomucoid (Gal d 1) remain the most widely investigated markers for cow’s milk and hen’s egg allergy, respectively. Proposed decision points for casein-specific IgE span from 0.95 to 14.10 kU/L [40,133,139,140,141,143,144], while ovomucoid-specific IgE thresholds range from 0.10 to 4.63 kU/L [33,40,67,145,146]. Given this variability, we used receiver operating characteristic (ROC) curve analysis to identify sIgE cutoff values and assess their diagnostic utility stratified by prior anaphylaxis.

For CMP, cutoff sIgE levels of 5.05 kU/L (CMP extract) and 64.10 kU/L (casein) were established for patients with a history of anaphylaxis to cow’s milk, whereas thresholds of 30.40 kU/L (CMP extract) and 18.80 kU/L (casein) were identified in those without prior anaphylaxis. For HEWP, cutoff sIgE levels of 5.24 kU/L (HEWP extract) and 3.59 kU/L (ovomucoid) were found in patients with a history of anaphylaxis to egg white, compared with 18.90 kU/L (HEWP extract) and 13.30 kU/L (ovomucoid) in those without such history. Similar associations were discovered in a work by Perry et al. [147], where cutoff sIgE levels differed in patients stratified by a clear history of reaction to the specific food allergen. Considering prior anaphylaxis sIgE marker type improve the prediction of CMP and HEWP OFC outcomes and enhance sIgE diagnostic utility. Delli Colli et al. [148] demonstrated the value of such combined approach by showing that logistic multivariate models—incorporating log-transformed casein concentrations and history of prior epinephrine use—could improve the prediction of OFC outcomes in pediatric patients.

Among patients without previous anaphylaxis to CMP, casein-specific IgE demonstrated the highest diagnostic utility (AUC = 0.81), closely followed by CMP extract (AUC = 0.80). Dominguez et al. [143] reported casein-sIgE to have the highest utility in predicting outcomes of OFCs to baked milk. Similarly, Nieminen et al. [140] identified casein-sIgE as the strongest predictor of tolerance to heated milk. Notably, across all subgroups of our cohort, the predictive performance of allergen extract-specific IgE (CMP and HEWP) was comparable to that of component testing (casein and ovomucoid), suggesting that extract-based assays may be equally informative, confronting the divergent results in the literature [25,28,149,150]. For HEWP, higher thresholds in children without prior anaphylaxis further support the hypothesis of a higher tolerability threshold in this group. However, it is essential to note that sIgE testing, including CRD, provides only limited accuracy regarding the potential challenge outcome [40]. In a study by Cronin et al. [151], casein sIgE concentrations at diagnosis were raised in patients who failed to achieve natural tolerance to cow’s milk. However, allergic reactions, including anaphylaxis, were not predicted by raised sIgE concentrations. On the contrary, Dodi et al. [152] confirmed the utility of sIgE for casein to predict the severity of clinical manifestations in failed OFCs. A systematic review by Riggioni et al. [25] reported high specificity of sIgE to individual components for allergy diagnosis—casein-specific IgE (93%) and ovomucoid-specific IgE (91%/92%), respectively. However, the authors emphasized that the diagnostic performance of these tests varies between populations, similarly to findings from another systematic review by Maesa et al. [149] revealing variability in diagnostic utility of components of different foods. The role of CRD remains unclear, due to ambiguous study results, various methods, sample sizes and populations studied [28,145,153]. Consequently, practical sIgE cutoff points are difficult to define, as they are usually developed for a particular cohort, and may have limited predictive ability in other populations [153].

In our study, cutoff values were selected to ensure high specificity and, consequently, a strong safety profile of the procedure. This, combined with a relatively small sample size, might have resulted in partially higher thresholds than previously reported in the literature. Notably, the meta-analysis by Riggioni et al. [25] provides separate cut-off values for hen’s egg depending on the degree of heat processing (raw, cooked, and baked), whereas for cow’s milk, cutoffs are reported only for milk extract and for casein-specific IgE, with no distinct thresholds proposed for baked milk challenges. This reflects the limited and heterogeneous evidence base for baked milk-specific serological cut-offs. In this context, our findings regarding casein-specific IgE and cow’s milk challenges align with the available literature, while the higher cutoffs observed in our cohort likely reflect the predominance of baked milk challenges and the focus on OFC safety rather than prediction of tolerance to raw milk. Our cohort comprises a highly allergic population, primarily undergoing baked food challenges, with sIgE concentrations exceeding those usually observed in patients considered for OFCs. The presented sIgE cutoff points do not pertain to the acquisition of food tolerance per se, but rather to the decision to safely perform an OFC under controlled conditions. Each patient warrants individualized assessment, including consideration of all factors that may affect the OFC outcome, particularly those related to previous history of anaphylaxis.

### 4.4. Strengths and Limitations

The main strengths of this study are related both to its setting and its design. Oral food challenges were performed in a tertiary pediatric allergy center, where children are followed longitudinally and each case undergoes detailed clinical assessment before being qualified for an OFC. In addition, we incorporated component-resolved diagnostics into routine evaluation, which provides a more refined characterization of food allergy than conventional testing. To our knowledge, this is one of the first studies to analyze OFC outcomes separately in children with and without a previous history of anaphylaxis to the culprit food, using extensive statistical comparisons between these two cohorts. This strategy allows for better risk stratification when planning OFCs in different patient subgroups, with the potential to improve procedural safety and to contribute meaningful data to the understanding of food allergy in childhood.

This study had several limitations. First, it relied on open OFCs rather than double-blind, placebo-controlled food challenges, which are considered the gold standard. However, open OFCs are widely accepted in clinical practice, particularly in young children. Because few subjective symptoms were reported and patients with inconclusive results were excluded from the statistical analysis, the impact of this limitation is likely minimal. Second, this was a retrospective, single-center study based on preexisting data, which may have introduced selection bias. We included OFCs performed from 2014 onward, a period during which CRD for FA (including specific IgE to casein and ovomucoid) was not yet routinely implemented in our clinical practice. As a result, CRD data were unavailable for a subset of patients, leading to discrepancies in the number of children with CRD results compared to those with extract-based sIgE measurements (CMP and HEWP). Third, the overall sample size was relatively small. Pediatric OFCs are resource-intensive and time-consuming, and the diversity of allergens combined with natural tolerance development complicates enrollment. Additionally, some patients and families decline OFCs due to fear of reactions and prefer continued avoidance. This may create a selection bias between children eligible for the procedure and those who ultimately undergo it. Consequently, many cases are managed or resolved without an OFC, contributing to the limited sample size. Another limitation of this study was the lack of systematic skin prick test (SPT) data. In many patients, SPT was not performed due to a history of severe anaphylactic reactions or the presence of moderate-to-severe atopic dermatitis, which may compromise test feasibility and reliability. Therefore, the diagnostic approach was primarily based on serum-specific IgE and component-resolved diagnostics.

## 5. Conclusions

In this study, we aimed to compare children with and without a prior history of anaphylaxis to the challenged food undergoing OFCs to CMP and HEWP, as well as to characterize pre-existing differences between these groups. We also sought to identify predictors of OFC failure stratified by anaphylaxis history. Our findings indicate that these two patient populations differ in several clinically relevant aspects, with differences in sIgE values and reactive doses visible especially in children undergoing challenges to HEWP. Atopic dermatitis and asthma emerged as potential risk factors for OFC outcomes, with their impact varying according to the child’s history of anaphylaxis. The predictive performance of sIgE likewise differed between anaphylaxis groups, yielding distinct cutoff points. Among the evaluated markers, casein-sIgE demonstrated the highest diagnostic accuracy in children without previous severe reactions to CMP. Although a prior history of anaphylaxis was associated with increased allergen sensitivity, it might not predict a higher risk of severe reactions during supervised OFCs. Nevertheless, a background of anaphylaxis remains an important consideration when selecting children for CMP and HEWP challenges, as it delineates distinct risk profiles and informs individualized risk assessment for OFC failure. Incorporating these considerations into clinical practice may enhance OFC safety and improve diagnostic accuracy. Finally, our study indicates that, in children without a prior history of anaphylaxis to the challenged food, HEWP OFCs might be initiated at higher starting doses than in those with previous anaphylaxis, without compromising procedural safety. The findings of this study may contribute to clinical practice, as a history of anaphylaxis has previously been investigated as a general risk factor for OFC failure without separate analysis of distinct risk factor profiles in patient groups stratified by the presence of prior anaphylaxis.

**Clinical take-home points:**

- A prior history of anaphylaxis might not increase the risk of severe reactions during supervised oral food challenges but is associated with higher allergen sensitivity and lower reactive doses.
- Predictors of oral food challenge failure differ by anaphylaxis history: atopic dermatitis is more relevant in children without prior anaphylaxis, while asthma in those with previous systemic reactions.
- Casein-specific IgE concentration is the most accurate serological predictor of cow’s milk challenge outcome in children without a history of anaphylaxis.

## Figures and Tables

**Table 1:** Characteristics of the study population.

Feature	Value
**Number of patients**	192 *
**Median age [IQR], (years)**	4.75 [3.32–6.82]
**Gender, *n* (%)**	
Male	127 (66.1%)
Female	65 (33.9%)
**Allergen challenged in the OFC, *n* (%)**	
Cow’s milk protein (CMP)	106 (55.2%)
Hen’s egg white protein (HEWP)	86 (44.8%)
**Median time from food consumption to reaction occurrence [IQR], (minutes)**	90.0 [60.0–120.0]
**Accompanying atopic diseases**	
Multi-food allergy	147 (76.6%)
Atopic dermatitis	120 (62.5%)
Asthma	97 (50.5%)
**Family history of atopy**	85 (44.3%)
**History of anaphylaxis to the challenged food ^#^**	110 (57.3%)
Grade 2	39 (35.5%)
Grade 3	34 (30.9%)
Grade 4	35 (31.8%)
Grade 5	2 (1.8%)

* including six patients with inconclusive OFC results; ^#^ applicable only to patients with a history of anaphylaxis to the challenged food. OFC—oral food challenge; CMP—cow’s milk protein; HEWP—hen’s egg white protein; IQR—interquartile range.

**Table 2:** Population characteristics by history of anaphylaxis to the challenged food.

Feature	History of Anaphylaxis to the Challenged Food * (*n* = 110)	No History of Anaphylaxis to the Challenged Food * (*n* = 82)	*p*
**No. of failed OFCs**	39 (35.5%)	23 (28.1%)	0.46
**Median age [IQR], (years)**	4.57 [2.96–6.87]	5.04 [3.70–6.78]	0.45
**Gender, *n* (%)**			
Male	69 (62.7%)	58 (70.7%)	0.25
Female	41 (37.3%)	24 (29.3%)	
**Allergen challenged in the OFC, *n* (%)**			
Cow’s milk protein (CMP)	70 (63.6%)	36 (43.9%)	**0.01**
Hen’s egg white protein (HEWP)	40 (36.4%)	46 (50.1%)	
**Median time from food consumption to reaction occurrence [IQR], (minutes)**	90.0 [65.0–150.0]	90.0 [50.0–120.0]	0.67
**Accompanying atopic diseases**			
Multi-food allergy	81 (73.6%)	66 (80.5%)	0.27
Atopic dermatitis	62 (56.4%)	58 (70.7%)	**0.04**
Asthma	60 (54.6%)	37 (45.1%)	0.20
**Family history of atopy**	52 (47.3%)	33 (40.2%)	0.33

* including patients with inconclusive OFC results. OFC—oral food challenge; IQR—interquartile range; CMP—cow’s milk protein; HEWP—hen’s egg white protein. Statistically significant *p* values (*p* < 0.05) are in bold.

**Table 3:** Cumulative reactive dose expressed in grams of allergenic protein in failed CMP and HEWP OFCs by history of anaphylaxis to the challenged food.

	N	CMP (g)	N	HEWP (g)	*p* Value
**History of anaphylaxis to the challenged food**	25	0.41 [0.13–1.09]	14	0.44 [0.29–1.02]	0.66
**No history of anaphylaxis to the challenged food**	7	0.54 [0.14–1.12]	16	1.17 [0.51–2.34]	0.15
***p* value**		0.45		**0.03**	

Data are presented as median and [interquartile range]. N—sample size in each group. CMP—cow’s milk protein; HEWP—hen’s egg white protein; Statistically significant *p* values (*p* < 0.05) are in bold.

**Table 4:** Influence of various factors on the OFC outcome by mean logistic regressions in patients with (N = 106) and without a history of anaphylaxis to the challenged food (N = 80).

Variable	History of Anaphylaxis (N = 106)		No History of Anaphylaxis (N = 80)	
	β (95% CI)	*p*	β (95% CI)	*p*
**Asthma**	0.83 (−0.03, 1.69)	0.05	−0.50 (−1.61, 0.62)	0.38
**Atopic dermatitis**	−0.44 (−1.29, 0.42)	0.32	**1.80 (0.34, 3.26)**	**0.02**
**Family history of atopy**	−0.14 (−0.97, 0.69)	0.74	−1.11 (−2.28, 0.06)	0.06
**Multi-food allergy**	0.82 (−0.27, 1.91)	0.14	1.59 (−0.10, 3.27)	0.07

β—regression coefficient; CI—confidence interval. Statistically significant *p* values (*p* < 0.05) are in bold.

**Table 5:** Specific IgE concentrations for passed and failed cow’s milk OFCs in patients with and without a history of anaphylaxis to the challenged food.

	CMP-Specific IgE [kU/L]					Casein-Specific IgE [kU/L]				
	N	Passed	N	Failed	*p*	N	Passed	N	Failed	*p*
**History of anaphylaxis to the challenged food**	41	6.19 [2.54–20.20]	23	19.40 [6.71–58.10]	**0.03**	33	4.99 [0.80–17.30]	20	10.99 [4.37–91.00]	0.05
**No history of anaphylaxis to the challenged food**	28	4.96 [1.25–21.55]	5	30.43 [30.40–45.00]	**0.04**	26	2.75 [0.53–8.90]	5	30.95 [18.80–37.10]	**0.03**
***p***		0.34		0.81			0.36		0.76	

Data are presented as median and [interquartile range]. N—sample size in each group. CMP—cow’s milk protein. Statistically significant *p* values (*p* < 0.05) are in bold.

**Table 6:** Specific IgE concentrations for passed and failed hen’s egg white OFCs in patients with and without a history of anaphylaxis to the challenged food.

	HEWP-Specific IgE [kU/L]					Ovomucoid-Specific IgE [kU/L]				
	N	Passed	N	Failed	*p*	N	Passed	N	Failed	*p*
**History of anaphylaxis to the challenged food**	23	3.26 [1.21–6.69]	12	11.20 [4.81–33.03]	**0.01**	19	1.67 [0.01–3.62]	9	10.30 [3.59–16.90]	**0.02**
**No history of anaphylaxis to the challenged food**	27	7.72 [3.86–20.30]	13	27.00 [11.30–39.00]	**0.03**	19	7.35 [1.91–14.95]	9	16.60 [5.16–24.20]	0.09
***p***		**0.02**		0.50			**0.04**		0.60	

Data are presented as median and [interquartile range]. N—sample size in each group. HEWP—hen’s egg white protein. Statistically significant *p* values (*p* < 0.05) are in bold.

## Data Availability

Data are not openly available due to ethical/privacy restrictions as permission for data sharing was not provided by the participants or the Ethical Committee.

## Supplementary Materials

The following supporting information can be downloaded at https://www.mdpi.com/article/10.3390/nu18020302/s1, Table S1: Characteristics of CMP and HEWP groups.

## Abbreviations

The following abbreviations are used in this manuscript:

FA	food allergy
EAACI	European Academy of Allergy and Clinical Immunology
CMP	cow’s milk protein
HEWP	hen’s egg white protein
sIgE	specific IgE
CRD	component-resolved diagnostics
BAT	basophil activation test
MAT	mast cell activation test
EDN	eosinophil-derived neurotoxin
OFC	oral food challenge
OIT	oral immunotherapy
FPIES	food protein-induced enterocolitis syndrome
ROC	receiver operating characteristic
AUC	area under the curve
AD	atopic dermatitis
