# Supplementary material for: Does Previous Anaphylaxis Determine Differences Between Patients Undergoing Oral Food Challenges to Cow’s Milk and Hen’s Egg?
Source: Nutrients. 2026 Jan 18;18(2):302. doi: 10.3390/nu18020302 (PMC12844613; doi:10.3390/nu18020302)
Supplement: Supplementary file 1 [file nutrients-18-00302-s001.zip › nutrients-4084388-supplementary.pdf]

Table S1. Characteristics of CMP and HEWP groups.

Feature	Trigger		p
	**CMP* (n=106)**	**HEWP* (n=86)**	
**No. of failed OFCs**	32 (30.2%)	30 (34.9%)	0.40
**Median age [IQR], (years)**	4.62 [3.16-6.23]	5.08 [3.45-6.92]	0.40
**Gender, n (%)**			
Male	71 (67.0%)	56 (65.1%)	0.79
Female	35 (33.0%)	30 (34.9%)	
**Median time from consumption to reaction occurrence [IQR], (minutes)**	90.0 [67.5-135.0]	90.0 [60.0-120.0]	0.62
**Accompanying atopic diseases**			
Multi-food allergy	79 (74.5%)	68 (79.1%)	0.46
Atopic dermatitis	60 (56.6%)	60 (69.8%)	0.06
Asthma	57 (53.8%)	40 (46.5%)	0.32
**Family history of atopy**	48 (45.3%)	37 (43.0%)	0.75
**Previous history of anaphylaxis to food challenged in the OFC**	70 (66.0%)	40 (46.5%)	**0.01**

*including patients with inconclusive OFC results. CMP—cow's milk protein; HEWP—hen's egg white protein; OFC – oral food challenge; IQR – interquartile range. Statistically significant p values ($p < 0.05$) are in bold.
